# Assessing the prevalence and effect of adverse drug reactions among patients receiving first line anti-tubercular medicines in the Tamale Teaching Hospital, Ghana

**DOI:** 10.11604/pamj.2021.38.191.24301

**Published:** 2021-02-19

**Authors:** Anthony Amalba, Amos Adapalala Bugri

**Affiliations:** 1 School of Medicine and Health Sciences, University for Development Studies, Tamale, Ghana,; 2 Pharmacy Directorate, Tamale Teaching Hospital, Tamale, Northern Region, Ghana

**Keywords:** Adverse drug reactions, tuberculosis, adherence, anti-tuberculosis therapy, occurrence, medication, Tamale

## Abstract

**Introduction:**

tuberculosis (TB) remains a global major health problem, especially in developing countries. Although treatment regimen for TB has been highly effective, treatment-related adverse effects account for significant morbidity leading to reduced effectiveness of therapy and high default rate. This study evaluated the nature and occurrence of Adverse Drug Reactions (ADRs) in patients receiving first line antitubercular therapy (ATT) in Tamale Teaching Hospital (TTH) and its effects on adherence.

**Methods:**

the study was a cross-sectional study for a period of six months. A total of 66 participants who were on first line antituberculotic therapy consented for the study. Data was collected using a questionnaire and analysed using SPSS version 22.0.

**Results:**

about 77% (n=51) of the participants had experienced ADRs. Gastrointestinal symptoms were the most commonly reported symptoms of ADR (80%, n=41). Regarding adherence, over half (51.0%, n=26) said the occurrence of the Adverse Drug Reaction had affected the manner in which they take their medications. Of these, 84.6% (n=22) of the participants indicated that they skipped/missed their medications and 15.4% stopped the medication entirely. About 39.2% (n=20) reported ADRs to a healthcare practitioner and 60.8% did not. All the reported cases were managed by a health practitioner using another medication.

**Conclusion:**

the study showed that ADRs are common among patients receiving first line ATT. Gastrointestinal tract (GIT) related ADRs were the most common. Adherence to first line antitubercular therapy is poor as a result of adverse drugs reaction.

## Introduction

Tuberculosis (TB) remains a global major health problem, especially in developing countries. The World Health Organization (WHO) in 1993 declared TB a global emergency in recognition of the growing importance of TB as a public health problem. In August 2005, TB was declared an African Emergency. About one-third of the world´s population is infected with *Mycobacterium tuberculosis*. According to WHO in 2018, an estimated 10 million people fell ill with TB worldwide. Five point seven (5.7) million men, 3.2 million women and 1.1 million children. There were cases in all countries and age groups. But TB is curable and preventable. Deaths from TB account for 25% of all avoidable deaths in developing countries. Some 95% of TB cases and 98% of TB deaths occur in developing countries. Of cases in developing countries, 75% are in the economically productive age group (15-50 years old). HIV is fuelling the TB epidemic. In the year 2009, an estimated 11-13% of incident cases were HIV positive. Of people infected with both HIV and *M. tuberculosis*, over 50% will become sick with TB during their lifetime; 10% will become sick per year [1]. Thus, the prevalence of HIV in a community has an important effect on the incidence of TB. Without treatment and in the absence of HIV infection, 50% of patients with pulmonary TB will die within 5 years, and 25% will remain sick with chronic, infectious TB [2].

It is estimated that Ghana has 86 smear positive pulmonary TB cases per 100,000 population and 106 of all types of TB cases per 100,000 population per year [3-5]. This means that with a population of about 28 million we should expect about 33,033 TB cases annually. However, only about 15,800 cases were reported in this country in 2011 of whom 50% were smear positive cases [6]. Tuberculosis (TB) is caused by bacteria *(Mycobacterium tuberculosis)* that most often affects the lungs but can affect other organs of the body [3]. In 2014, there was an estimated 9.6 million new cases reported to WHO and 1.5 million deaths around the world. In Morocco, TB remains the leading cause of serious illness with an estimated incidence of 106 (97-105) per 100,000 [4]. However, in Ghana following the national prevalence survey in the year 2013, estimated prevalence rate was 282 per 100,000 population and estimated mortality rate of 36 per 100,000 according to the National Tuberculosis Programme (NTP).

The standardized empirical treatment as recommended by WHO consists of two months intensive phase, during which period patients take drugs under direct observation by a health staff, following that is a continuation phase of four months for new TB cases. The re-treatment cases provide three months of intensive phase and five months of continuation phase for susceptible TB. The drugs used for the initial empiric treatment of new TB cases consists of isoniazid, rifampin, pyrazinamide, and ethambutol which are administered as a four-drug regimen daily for two months. There is drastic reduction in bacteria load as they kill the tubercle bacilli rapidly. Infectious patients are rendered non-infectious in about two weeks of therapy. The continuation phase follows immediately after the intensive phase with daily administration of isoniazid and rifampin for four more months [7, 8]. Although the treatment regimen has been highly effective, treatment-related adverse effects including hepatotoxicity, skin reactions, gastrointestinal and neurological disorders account for significant morbidity leading to reduced effectiveness of therapy and high default rate.

Adverse Drug Reactions (ADRs) is a response to drug which is noxious and unintended and which occurs at doses normally used as prophylaxis, diagnosis or therapy of diseases for modification of physiological function [9]. These ADRs could pose a serious challenge to the completion of the regimen. The kind of ADRs that are experienced by these patients needs to be investigated and a profile of the reaction can be ascertained at our treatment site. Adverse drug reactions (ADRs) can often cause significant morbidity among individuals on anti-tubercular therapy, occasionally leading to mortality. Treatment-limiting drug toxicities can complicate the management of TB by impairing patients´ adherence to treatment and thus lead to inferior clinical outcomes and higher cost to the public health system.

The standard treatment for TB is very effective but has also significant adverse drug reactions which sometimes may cause patients to stop taking their medication or could even lead to hospitalization [10, 11]. A total of about 200 clients were registered and put on first line medications in the Tamale Teaching Hospital in 2016. The default rate for 2017 was 12.5% which is high compared to the national acceptable figure of about 1%, with a total of 25% as death rate [6]. In Ghana, little research has been conducted to ascertain the effects of ADRs on treatment outcomes like default. Considering the low treatment success rate of 62.5 and high default rate of 12.5 for the year 2017, it is important to investigate the causes of this low success and high default rates on the use of anti-TB medicines. Could adverse drug reaction be part of the reasons for this high default rate? This study therefore explores the effects of adverse drug reaction on patients receiving first-line anti-tubercular medicines at the Tamale Teaching Hospital and how the occurrence of these ADRs and their management affect adherence.

## Methods

**Study design:** a cross-sectional descriptive study was conducted at Tamale Teaching Hospital from 1^st^ October, 2017 to 30^th^ April, 2018.

**Setting:** we conducted this study in one of the sixteen administrative regions of Ghana, specifically northern region which has Tamale as its capital city and a population of over 2 million inhabitants. Tamale Teaching Hospital (TTH) is located in Tamale and it is a referral centre with a well-established chest clinic responsible for the diagnosis and treatment of TB. It is currently located on the eastern part of the Tamale Metropolis on the main hospital road. It has a land area of approximately four-hundred and ninety-thousand (490,000m^2^) square metres. The hospital was previously located at the Tamale Central Hospital in the heart of the main Tamale business town. The TTH was commissioned in 1974 as a regional hospital to serve as a referral centre for the much-needed medical services for the northern sector and also serve the people of Tamale and its environs. But later was transformed to a tertiary facility and teaching hospital in 2008. The hospital has a total bed capacity of 800 and receives about 100,000 outpatients´ visits at the outpatient department and records or treats an average of 200 TB patients annually.

**Questionnaire and participants:** all participants who visited the chest clinic of TTH and were on first line anti-tubercular therapy and consented for the study were recruited. Inclusion criteria for participants were: all patients who are on first line anti-tubercular therapy and registered on the TB register in the chest clinic. Exclusion criteria were: patients who have previously been exposed to anti-tubercular medication e.g. relapse, treatment failure and other categories were excluded from the study, patients who have been diagnosed of other chronic disease condition(s) in addition to TB and are taking other medication apart from the anti-tubercular medication, pregnant women on anti-tubercular medication and all those who did not consent to be part of the study.

The study team comprised of one pharmacist (the researcher), three pharmacist interns, two general nurses. One-hour orientation training was given to them on the rational of the study, selection of study participants and the study variables to be measured. The study was conducted for a period of seven months, between 1^st^ October, 2017 and 30^th^ April, 2018. The data was obtained from participants through the use of a questionnaire. Socio-demographic variables including sex, age, level of education, and employment status were collected on participants and retrieval of types and/or category of anti-tubercular therapy from their folder.

The questionnaire was piloted on a sample of ten participants to make sure they were comprehensible by the study participants. There were minor changes in the form editing the English. These ten participants were not included in the study. The ‘cola colored urine´ and ‘take after meals´ were some the options included in the questionnaire after the validation process. Ethical approval was granted by the Tamale Teaching Hospital Ethical Review Committee for all data collection procedures and processes of obtaining informed consent.

**Data analysis:** data collected was cleaned, coded and analyzed using SPSS version 22.0 (IBM Corp, N.Y, USA) to obtain descriptive data. Relevant tables and figures were created from the data to allow for easy analysis and interpretation. Frequency and percentage were used to determine the prevalence of ADRs among the patients on first line antitubercular therapy. Pattern of commonly experienced ADRs and whether ADRs caused participants to default treatment or not. Continuous variables such as age, was presented in ranges. Chi squared test was used to test whether the occurrence of ADRs affected adherence, with p-value < 0.05 was used to assess the level of significance.

## Results

**Figure 1:**
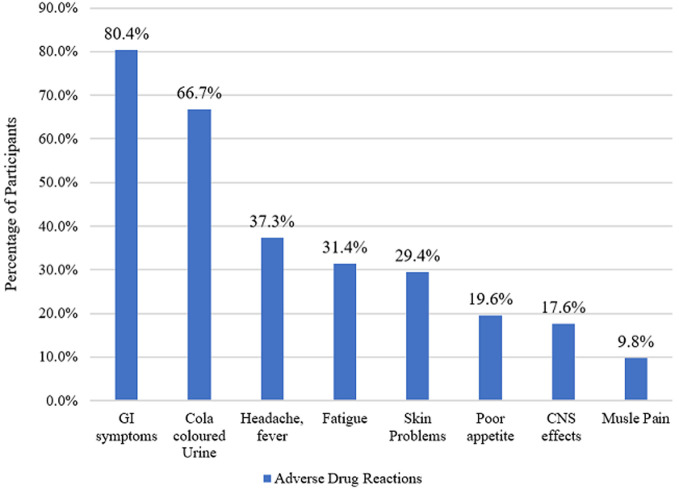
adverse reactions experienced by patients

A total of sixty-six (66) participants were recruited for this study from the Tuberculosis clinic of the Tamale Teaching Hospital. The average age was 46.4 years, with a minimum of 8 years and a maximum of 95 years. Majority of the participants (57.6%, n=38) were within the age group of 31 to 60 years. About 48.5% (n=32) of the study participants weighed between 40 to 54 kg. Both males and females were represented equally within the study with 50.0% (n=33) each. Of the 66 participants, most (60.6%, n=40) were married, and 1.5% divorced. Around 48.5% of respondents had no formal education with only 7.6% (n=5) having tertiary level of education. Most of the respondents (56.9%, n=37) were unemployed. Out of the 25 participants who were employed, 96.0% (n=24) had day jobs (Table 1). Out of the 29 respondents who were aware of possible ADRs, over 80.0% indicated gastrointestinal (GI) symptoms, 66.7% cola coloured urine (Figure 1).

**Table 1:** demographic characteristics

Variable	Frequency	Percentage
**Age**		
18 or less	6	9.1
19 - 30	10	15.2
31 - 60	38	57.6
61 and above	12	18.2
**Gender**		
Male	33	50.0
Female	33	50.0
**Marital status**		
Married	40	60.6
Single	13	19.7
Divorced	1	1.5
Separated	3	4.5
Widowed	9	13.6
**Educational qualification**		
None	32	48.5
Primary	15	22.7
*JHS/MSLC	10	15.2
*SHS	4	6.1
Tertiary	5	7.6
**If tertiary, specify**		
Polytechnic	4	80.0
University	1	20.0
**Religion**		
Christian	14	21.2
Muslim	52	78.8
**Employment status**		
Employed	25	37.87
Unemployed	37	56.06
N/A	4	6.06
**If employed, time of work**		
Day	24	96.0
Night	1	4.0
**Weight (Kg)**		
20 and below	1	1.5
21 - 29	5	7.6
30 - 39	10	15.2
40 - 54	32	48.5
55 and above	18	27.3

MSLC: Middle School Leaving Certificate; JHS: Junior High School; SHS: Senior High School.

**Adverse drug reaction occurrence and adherence to anti-tubercular medication:** all patients (100.0%, n=66) on anti-tubercular medication indicated they took their medication. Almost all patients (97.0%, n=64) said they took their medication daily except 2 patients (3.0%) who were not taking their daily doses due to side effects and busy schedule. Over half (51.5%, n=34) of participants indicated that they had ever missed their dose. Of the 34 respondents who admitted to missing a dose, 97.1% (n=33) indicated that they took the medication as soon as they remembered. A significant number of participants adhered to their daily medication intake despite ADR (p<0.001) (Table 2).

**Table 2:** compliance and adherence with anti-tubercular therapy

Variable	Frequency	Percentage	p-value
**Do you take your medication?**			
Yes	66	66	
No	0	0	
**If yes, how often?**			
Daily	64	97	
Not daily	2	3	
**If no, why?**			
Busy schedule	1	50	
Side effects	1	50	
**Adherence**			
Yes	34	51.5	
No	32	48.5	
**Experienced ADR**			
Yes	51	77.3	
No	15	22.7	
**ADR effects on adherence**			
Yes	26	51	
No	25	49	
**If yes, in what way?**			
Miss/skip doses	22	84.6	
Stop taking medication	4	15.4	
**Adherence to medication daily**			
Yes	49	74.2	0.0001
No	15	22.7	

About 77.3% (n=51) of study participants indicated that they had ever experienced ADRs. Out of these 51 participants, over half (51.0%, n=26) said the occurrence of the adverse drug reaction had affected the manner in which they took their medications, whiles 21 (84.6%) said the ADRs made them to skip or miss medication, 4 (15.4%) said they stopped taking their medication as shown in Table 2.

All the 17 reported cases (73.9%) were managed by a health practitioner. About 82.4% (n=14) of patients who had their cases managed indicated that they were satisfied with the management of ADRs received. The majority (90.9%, n=60) of patients attending TB clinic were counselled on anti-tubercular medications. Of those who were counselled, 78.3% (n=47) of them were counselled by a nurse (Table 3).

**Table 3:** adverse drug reaction management and counselling

Variable	Frequency	Percentage
**Reported ADR**		
Yes	17	73.9
No	6	26.1
**If no, why?**		
Lack of expertise	4	66.7
Financial constraints	1	16.7
Others	1	16.7
**ADR was managed by healthcare practitioner**	17	100
**How was your ADR managed?**		
Therapy was stopped	0	0
Therapy was changed	1	5.9
Medications were prescribed to manage it	12	70.6
Others	4	23.5
**Satisfied with the management of ADR**		
Yes	14	82.4
No	3	17.6
**If not satisfied, why?**		
Poorly managed	1	33.3
Felt there were better options	2	66.7
**Received counselling on anti-tubercular medications?**		
Yes	60	90.9
No	6	9.1
**Who counselled you on anti-tubercular medications?**		
Doctor	13	21.7
Nurse	47	78.3
**Understanding the counselling provided**		
Yes	54	90
No	6	10
**If not, why**		
Time was short	1	14.3
No room for feedback	1	14.3
Too much information	3	42.9
Language barrier	1	14.3
Long and boring	1	14.3
**Knowledge of possible adverse drug reaction**		
Yes	31	52.5
No	28	47.5

## Discussion

This was the first, hospital-based study to assess the prevalence and effects of ADRs among patients receiving first-line anti-tuberculosis medications in the Tamale Teaching Hospital and the northern sector as whole in Ghana. Among the various problems associated with TB, the toxicity associated with anti-tubercular therapy (ATT) drugs was still a major concern. Our study found that majority of the study participants indicated that they experienced some forms of adverse drug reactions (ADRs). Though all patients indicated they take their medication, 51% experienced ADR and that the occurrence of ADR has affected the way they take their medication either by missing or skipping doses or stop taking medication entirely. One of the main reasons for treatment interruption among TB patients is adverse drug reactions [12]. It was also obvious from this study that patients who experienced ADR either skipped doses of ATT or stopped completely taking the antitubercular drugs. This correlates with the consistently high lost to follow-up or default rates in TTH, as reported in programme indicators [6] *(NTP manual, 2012)*. It may worsen the already difficult war against the antimicrobial resistance battle. The default rate which was increasing among clients could also be attributed to these ADRs. It was obvious that the ADRs affected the way and manner the patients took their drugs.

The ADRs due to TB drugs can therefore not be underestimated considering the number who actually experienced this ADRs. This is consisted with literature as has been reported in similar studies conducted in India and Russia [13, 14]. Identifying the causes of ADRs is an important responsibility of the healthcare professionals and can prevent the occurrence of similar ADRs in future. Our patients were on the combined fixed dose regimen and identifying the actual molecule causing the adverse reactions was a challenge. But from literature, the GIT ADR experienced suggests that the Rifampicin and Isoniazid may be culprits and cola or orange coloured urine could be as a result of the Rifampicin.

One of the main reasons for treatment interruption among TB patients is adverse drug reactions [12]. The burden of TB ADRs in the treatment of TB cannot be overemphasised. Compliance and adherence with anti-tubercular therapy is very key in achieving good clinical outcomes of patients undergoing anti-tubercular therapy. Even though a significant (74.2%, p=0.001) number of patients indicated that they adhered to daily medication, the remaining number that do not adhere to medication as a result of ADR is a worry to the National Tuberculosis Programme in achieving complete compliance and adherence to anti-tubercular therapy. It is one of the barriers to successful TB treatment and affects most TB programmes in developing countries like Ghana. In the TB programme, most patients who experience ADRs default their treatment and resume at a later date causing increase in the resistance to TB treatment. Some clients even stopped treatment entirely posing danger to themselves and their community as whole.

Majority of patients who experienced ADR reported to a healthcare practitioner and this was managed mostly by prescribing different medication to minimise the ADR. The finding and evaluation of ADRs in hospitals are required because of the possibility of identification of severe reactions, reactions of new drugs, increased frequency of known reactions, unknown effects, identification of the risk factors and possible dissemination of information among clinicians and health professionals [15]. Several studies have been conducted to describe the ADR profile found in hospitals, evaluating medicaments, therapeutic classes and demographic data of affected patients, medications concomitantly used for a certain person, type of ADR, affected organs, severity and causality relation [10, 16, 17]. In some countries, pharmacovigilance is based on the spontaneous reporting aimed at ADR discovery after commercialization [15].

However, a major drawback of this model is that only a small part of all ADRs is reported [18]. Previous reports showed that several factors were associated with under-reporting such as ignorance (only severe ADRs need to be reported), hesitancy (fear of appearing ridiculous for reporting merely a suspected ADR), lethargy (e.g. lack of interest or time), indifference (one case from an individual practitioner does not contribute to medical knowledge), insecurity (causality between a drug and an adverse event is hard to determine) and complacency (only safe drugs are on the market) [15, 18]. This study also saw just a few reporting their ADR to health professional for management.

One important finding in this study was that majority of the patients were counselled generally before treatment. It was possible that most patients received counselling on the importance of taking their medication on a regular basis. From this study, though 90.9% patients received counselling on anti-tubercular medications, 47.5% were not counselled on the possible adverse drug reaction on anti-tubercular therapy. It is the responsibility of the health care professionals to counsel the patients regarding ADRs. It was also observed that most of the counselling in this study was done by the directly observed treatment (DOTS) corner nurses before initiation of ATT. There will be the need to include pharmacists who have a deeper knowledge on drugs and their adverse effects to be included in the counselling of patients on anti-tubercular therapy. This may improve the knowledge of these clients about ADR and possible prevention of their occurrence.

Again, the training of DOT corner nurses and physicians on counselling on ADR could also improve clients´ knowledge on ADR. Out of 51 patients who had ever experienced ADRs, 39.2% reported the ADR to a healthcare practitioner and 60.8% did not report the incident. Eighty-three-point nine percent (83.9%) of patients who did not report the reaction to a health practitioner indicated that they didn´t consult anybody while 16.1% said they consulted others (friends, peers, relatives and colleagues) as shown in Table 3. These ADRs were mild and hence could be the reason why they did not bother to report to any health worker. The lack of counselling about ADRs, the fear of being stigmatized by the public could be some of the reasons why some of the clients did not even report the ADR to anybody even though they confirmed experiencing the ADR. Lack of confidence in the health professional or out of fear of the white coat could be the reason for not reporting these ADRs experienced. And those who did not report to anyone probably knew that the ADR were expected and hence decided to contain them.

**Limitations of study:** the present study has some limitations. This study was conducted in a single referral teaching hospital making it difficult to generalize our findings. However, the findings are in agreement with similar studies conducted in both developed and developing countries. It also serves as a baseline for further studies in other referral hospitals in Ghana. This is also a cross-sectional study and makes it difficult to determine the direction of the association.

## Conclusion

From the study, adverse drug reactions are common among patients receiving first line anti-tubercular drugs. The study revealed about 77% of the participants experienced ADRs. This study also demonstrated that GIT related ADRs were the most common ADR (about 80%) experienced by patients receiving first line anti-tubercular therapy. The occurrence of these adverse drug reactions affected the way and manner these patients actually took their medication. The adverse drug reactions experienced (about 60%) were not reported to any health worker and hence were not managed by a health professional. The clients were not offered counselling on ADRs related to TB according to the study. However, almost all the patients received some form of general counselling by DOTs corner nurses before the initiation of therapy.

### What is known about this topic

- Tuberculosis remains a leading cause of serious illness especially developing countries;
- Tuberculosis is curable and preventable;
- Treatment regimen for tuberculosis is highly effective, though with treatment related adverse effects.

### What this study adds

- We found in our study that adverse drug reaction is a leading cause of default rate or noncompliance leading either to skipping of doses or complete halt of taking anti-tuberculosis drug;
- Majority of TB patients received counselling before treatment and counselling was done mainly by nurses and not pharmacists;
- Lack of counselling and fear of stigmatization are reasons patients do not report adverse drug reaction.
